# Supplementary material for: Taking account of asymptomatic infections: A modeling study of the COVID-19 outbreak on the Diamond Princess cruise ship
Source: PLoS One. 2021 Mar 16;16(3):e0248273. doi: 10.1371/journal.pone.0248273 (PMC7963096; doi:10.1371/journal.pone.0248273)
Supplement: S1 File — (PDF) [file pone.0248273.s001.pdf]

```
##################### eAppendix ########################################
########################################################################
#  R code for the paper with
# title: Taking Account of Asymptomatic Infections ¡V
#        A Modeling Study on the COVID-19 Outbreak
#        on the Diamond Princess Cruise Ship
# serial interval= 6 days
########################################################################

########### Data #######################################################
# data on the Number of individuals testing positive from Table in
#  Mizumoto K, Kagaya K, Zarebski A, Chowell G. Estimating the
#        asymptomatic proportion of coronavirus disease 2019 (COVID-19)
#        cases on board the Diamond Princess cruise ship, Yokohama,
#        Japan, 2020. Eurosurveillance 2020;25(10).
#        doi: 10.2807/1560¡V7917.ES.2020.25.10.2000180.
#
# We set Jan 20 as day 0. Then Jan 21 is day 1, Feb 3 is day 14
# Feb 6 is day 17,  Feb 7 - 10 are days 18 - 21, Feb 12 is day 23


days <- c(1,  14,  17, 18, 19, 20, 21, 23, 24, 26, 27, 28, 29, 30)
cases <- c(1, 10, 10,  41,  3,   6,  65,  39, 44, 67, 70, 99, 88, 79)
cumcases<- cumsum(cases)
# [1]   1  11  21  62  65  71 136 175 219 286 356 455 543 622
#
#*######################################################################
### based on field briefing,
# National Institute of Infectious Disease. Field briefing: Diamond
# Princess COVID-19 cases.
# https://www.niid.go.jp/niid/en/2019-ncov-e/9407-covid-dp-fe-01.html
#
# 34 cases before 2/6, adjust cases[2:4]
########################################################################
cases[2]<- cases[2]+7
cases[3]<- cases[3]+7
cases[4]<- cases[4]-14
cumcases<- cumsum(cases)

#> cumcases
# [1]   1  18  35  62  65  71 136 175 219 286 356 455 543 622

########################################################################
#For those dates that Y, the number of new COVID-19 cases, was not
# reported, linear interpolation was used.
#
#
dayseq<-seq(1, 30, by=1)
caseseq <- rep(1.0, length(dayseq))


caseseq[2:14] <- cases[2]/(days[2]-days[1])
caseseq[15:17] <- cases[3]/(days[3]-days[2])
caseseq[18:21]<- cases[4:7]
caseseq[22:23] <- cases[8]/(days[8]-days[7])
caseseq[24] <- cases[9]
caseseq[25:26] <- cases[10]/(days[10]-days[9])
caseseq[27:30] <- cases[11:14]

#> caseseq
# [1]  1.000000  1.307692  1.307692  1.307692  1.307692  1.307692  1.307692
# [8]  1.307692  1.307692  1.307692  1.307692  1.307692  1.307692  1.307692
# [15]  5.666667  5.666667  5.666667 27.000000  3.000000  6.000000 65.000000
# [22] 19.500000 19.500000 44.000000 33.500000 33.500000 70.000000 99.000000
# [29] 88.000000 79.000000

#round to integers
```

```
caseseq[6:8] <- 1
caseseq[9]<- 2

caseseq[10:12] <- 1
caseseq[13]<- 2

caseseq[14]<- 2

caseseq[15]<- 5
caseseq[16]<- 6
caseseq[17]<- 6

caseseq[22]<- 19
caseseq[23]<- 20
caseseq[25] <- 33
caseseq[26] <- 34

#> caseseq
# [1]  1  1  1  1  2  1  1  1  2  1  1  1  2  2  5  6  6 27  3  6 65 19 20 44 33
# [26] 34 70 99 88 79

######################################################################
#serial interval 6 days
#Then daily data were aggregated into 6-day intervals

sixdays=rep(1:5, each=6)
y=sapply(split(caseseq[1:30], sixdays), sum)

#> y
#  1   2   3   4   5
#  7   7  48 157 403
# represents 1/21-1/26, 1/27-2/1, 2/2-2/7, 2/8-2/13, 2/13-2/20

#
#case 1 at day 0
y<- c(1,y)
y[2]<- y[2]-1

#> y
#      1   2   3   4   5
#  1   6   7  48 157 403

names(y)[1] <- "0"

#> y
#  0   1   2   3   4   5
#  1   6   7  48 157 403

#cumulative cases
#include case 1 at t=0
cumy<- cumsum(y)[2:5]

#> cumy
#  0   1   2   3   4   5
#  1   7  14  62 219 622


#number of people at risk on board if infected is removed
N0=3712-cumy

#> N0
#   0    1    2    3    4    5
#3711 3705 3698 3650 3493 3090

######################################################################
#number of crew member 1045 + passenger 2666

# field brifing
[illegible]
```

```
# among 619 cases (=82 crew+ 537 passengers)
fc=82/619
fp= 537/619


# assumption (d)(i) in the paper from 2/8 assume that
# of all infected cases, 86.8%(=537/619) were passengers and
# 13.2% (=82/619) crew. We use these proportions to calculate
# the number of infected persons in each group

cumicrew=floor(fc*cumy[])

#> cumicrew
# 0  1  2  3  4  5
# 0  0  1  8 29 82


icrew=c(0, diff(cumicrew))
#infected crew, paper notation Ict
#> icrew
#    1  2  3  4  5
# 0  0  1  7 21 53

names(icrew)[1] <- "0"

#> icrew
# 0  1  2  3  4  5
# 0  0  1  7 21 53

# c0 is (crew-infected crew(removed))
# in the paper notation Ct is the number of crew members on board at time t

c0= rep(1045,6)-cumicrew

#> c0
#   0    1    2    3    4    5
#1045 1045 1044 1037 1016  963


# number of infected passengers (case 1 at day 0 included) at time t
# in the paper notation Ipt
ipger<- y-icrew
#> ipger
#  0   1   2   3   4   5
#  1   6   6  41 136 350


#pgr0 is number (passengers - infected passengers(removed)) on board
pgr0= c(rep(2667, 6))-cumsum(ipger)

#> pgr0
#   0    1    2    3    4    5
#2666 2660 2654 2613 2477 2127

######################################################################
########## Likelihood Function #########################################

llik.cb.new=function (beta, I, aratio, roomp)
{  # for serial-interval 6 days
   # I is number of new cases

    #index=1 is time 0  I[1]=1
    #likelihood 1/21-1/26, assumption (a)
    p1= 1 - exp(-beta*I[1]/(N0[1]+1))
    L= -sum(dbinom(I[2], N0[1], p1, log = TRUE) )

    #likelihood from 1/27-2/1  assumption (b)
    p2= 1 - exp(-beta*I[2]/N0[1])
```

```
    p3= 1 - exp(-beta*(I[2]+I[3])/N0[1])
    L = L - sum(dbinom(I[4], N0[3], p3, log = TRUE) )

    ####likelihood from 2/8-2/13
    # assumption (d)(ii)
    pcrew = 1 - exp(-beta * I[4]/(N0[3]))
    L = L -dbinom(icrew[5], c0[4], pcrew, log = TRUE)

    # Passengers stayed in cabins most of the time. Assume that
    # among infected passengers Ipt, the proportion of infections
    # that occurred in cabins is rp, and that the average occupancy
    # per cabin is 2.
    # assumption (d)(iii)
    ppger2=1 - exp(-beta * 0.5)
    ipger2=floor(ipger[5]*roomp)

    L = L -dbinom(ipger2, ipger[4], ppger2, log = TRUE)

    # The other (1 ¡V rp) proportion of infected passengers¡¦ cases
    # was possibly due to asymptomatic crew members who continued to work.
    # assumption (d)(iv)
    ppger = 1 - exp(-beta * aratio*icrew[4]/c0[4])
    ipger2rm=ipger[5] - ipger2  #remaing (1-rp) cases
    L = L -dbinom(ipger2rm, (pgr0[4]-ipger[4]), ppger, log = TRUE)


    #### likelihood from  2/14-2/19, analogous to 2/8-2/13
    # assumption (d)(ii)
    pcrew = 1 - exp(-beta *I[5]/(N0[4]))
    L = L -dbinom(icrew[6], c0[5], pcrew, log = TRUE)


    # assumption (d)(iii)
    ppger2=1 - exp(-beta * 0.5)
    ipger2=floor(ipger[6]*roomp)

    L = L -dbinom(ipger2, ipger[5], ppger2, log = TRUE)

    # assumption (d)(iv)
    ppger = 1 - exp(-beta * aratio*icrew[5]/c0[5])
    ipger2rm=ipger[6] - ipger2  #remaing (1-rp) cases
    L = L -dbinom(ipger2rm, (pgr0[5]-ipger[5]), ppger, log = TRUE)


    return(L)
}

######################################################################
####### modify some R-functions in Bjornstad (2018) ##################
# and use bbmle R-package
# Bolker B. bbmle: Tools for general maximum likelihood estimation.
# https://cran.r-project.org/web/packages/bbmle/.
#
######################################################################
################ roomp=rp=0.2 ########################################
################# aratio=0.4 #########################################
#percentages of infection in the same room
roomp<- 23/115


betacand = seq(0.1,15, by = 0.05)
ll = rep(NA, length(betacand))

# plot the likelihood and check maximum
for(i in 1:length(betacand)){
     ll[i] = llik.cb.new(beta = betacand[i],
     I = y, aratio=0.4, roomp=0.2)}
plot(ll ~ betacand, ylab = "Neg log-lik",
```

```
#> betacand[which.min(ll)]
#[1] 3.95

fit = mle2(llik.cb.new, start = list(beta = 3.95), fixed=list(aratio=0.4, roomp=0.2),
    method = "Brent", data = list(I = y), lower=1, upper=15)


summary(fit)
confint(fit)

# > summary(fit)
# Maximum likelihood estimation
#
# Call:
# mle2(minuslogl = llik.cb.new, start = list(beta = 3.95), method = "Brent",
#    fixed = list(aratio = 0.4, roomp = 0.2), data = list(I = y),
#    lower = 1, upper = 15)
#
# Coefficients:
#      Estimate Std. Error z value     Pr(z)
# beta  3.94418    0.16174  24.386 < 2.2e-16 ***
# ---
# Signif. codes:  0 ¡¥***¡¦ 0.001 ¡¥**¡¦ 0.01 ¡¥*¡¦ 0.05 ¡¥.¡¦ 0.1 ¡¥ ¡¦ 1
#
# -2 log L: 787.4827
#  > confint(fit)
#  Profiling...
#
#    2.5 %   97.5 %
#  3.635916 4.270077


################ roomp=rp= 0.2 ########################################
################# aratio=0.465 ########################################

betacand = seq(0.1,15, by = 0.05)
ll = rep(NA, length(betacand))

# plot the likelihood and check maximum
for(i in 1:length(betacand)){
     ll[i] = llik.cb.new(beta = betacand[i],
     I = y, aratio=0.465, roomp=0.2)}
plot(ll ~ betacand, ylab = "Neg log-lik",
     xlab = expression(beta))
betacand[which.min(ll)]

# > betacand[which.min(ll)]
# [1] 3.85

fit = mle2(llik.cb.new, start = list(beta = 3.85), fixed=list(aratio=0.465, roomp=0.2),
    method = "Brent", data = list(I = y), lower=1, upper=15)

summary(fit)
confint(fit)

#  > summary(fit)
#  Maximum likelihood estimation
#
# Call:
# mle2(minuslogl = llik.cb.new, start = list(beta = 3.85), method = "Brent",
#     fixed = list(aratio = 0.465, roomp = 0.2), data = list(I = y),
#     lower = 1, upper = 15)
#
# Coefficients:
#      Estimate Std. Error z value     Pr(z)
# beta  3.84087    0.15735  24.409 < 2.2e-16 ***
# ---
# Signif. codes:  0 ¡¥***¡¦ 0.001 ¡¥**¡¦ 0.01 ¡¥*¡¦ 0.05 ¡¥.¡¦ 0.1 ¡¥ ¡¦ 1
```

```
#  Profiling...
#
#    2.5 %   97.5 %
# 3.540946 4.157911


################ roomp=rp=0.2 ########################################
################# aratio=0.505 #######################################

betacand = seq(0.1,15, by = 0.05)
ll = rep(NA, length(betacand))

# plot the likelihood and check maximum
for(i in 1:length(betacand)){
     ll[i] = llik.cb.new(beta = betacand[i],
     I = y, aratio=0.505, roomp=0.2)}
plot(ll ~ betacand, ylab = "Neg log-lik",
     xlab = expression(beta))
betacand[which.min(ll)]

# > betacand[which.min(ll)]
# [1] 3.8

fit = mle2(llik.cb.new, start = list(beta = 3.8), fixed=list(aratio=0.505, roomp=0.2),
    method = "Brent", data = list(I = y), lower=1, upper=15)


summary(fit)
confint(fit)

# > summary(fit)
# Maximum likelihood estimation
#
# Call:
# mle2(minuslogl = llik.cb.new, start = list(beta = 3.8), method = "Brent",
#     fixed = list(aratio = 0.505, roomp = 0.2), data = list(I = y),
#     lower = 1, upper = 15)
#
# Coefficients:
#      Estimate Std. Error z value     Pr(z)
# beta  3.78004    0.15477  24.423 < 2.2e-16 ***
# ---
# Signif. codes:  0 ¡¥***¡¦ 0.001 ¡¥**¡¦ 0.01 ¡¥*¡¦ 0.05 ¡¥.¡¦ 0.1 ¡¥ ¡¦ 1
#
# -2 log L: 656.7847
# > confint(fit)
# Profiling...
#
#    2.5 %   97.5 %
# 3.485014 4.091871



################ roomp=rp=0.2 ########################################
################# aratio=0.6 #########################################

betacand = seq(0.1,15, by = 0.05)
ll = rep(NA, length(betacand))

# plot the likelihood and check maximum
for(i in 1:length(betacand)){
     ll[i] = llik.cb.new(beta = betacand[i],
     I = y, aratio=0.6, roomp=0.2)}
plot(ll ~ betacand, ylab = "Neg log-lik",
     xlab = expression(beta))
betacand[which.min(ll)]

# > betacand[which.min(ll)]
```

```
    method = "Brent", data = list(I = y), lower=1, upper=15)


summary(fit)
confint(fit)

# > summary(fit)
# Maximum likelihood estimation
#
# Call:
# mle2(minuslogl = llik.cb.new, start = list(beta = 3.65), method = "Brent",
#     fixed = list(aratio = 0.6, roomp = 0.2), data = list(I = y),
#     lower = 1, upper = 15)
#
# Coefficients:
#      Estimate Std. Error z value     Pr(z)
# beta  3.64324    0.14899  24.453 < 2.2e-16 ***
# ---
# Signif. codes:  0 ¡¥***¡¦ 0.001 ¡¥**¡¦ 0.01 ¡¥*¡¦ 0.05 ¡¥.¡¦ 0.1 ¡¥ ¡¦ 1
#
# -2 log L: 566.721
# > confint(fit)
# Profiling...
#
#    2.5 %   97.5 %
#  3.359223 3.943399
#



########### different roomp=rp=0.3 ####################################
################# aratio=0.4 ##########################################
roomp =0.3

betacand = seq(0.1,15, by = 0.05)
ll = rep(NA, length(betacand))

# plot the likelihood and check maximum
for(i in 1:length(betacand)){
     ll[i] = llik.cb.new(beta = betacand[i],
     I = y, aratio=0.4, roomp=0.3)}
plot(ll ~ betacand, ylab = "Neg log-lik",
     xlab = expression(beta))
betacand[which.min(ll)]

# > betacand[which.min(ll)]
# [1] 4.4

fit = mle2(llik.cb.new, start = list(beta = 4.4), fixed=list(aratio=0.4, roomp=0.3),
    method = "Brent", data = list(I = y), lower=1, upper=15)


summary(fit)
confint(fit)

# > summary(fit)
# Maximum likelihood estimation
#
# Call:
# mle2(minuslogl = llik.cb.new, start = list(beta = 4.4), method = "Brent",
#     fixed = list(aratio = 0.4, roomp = 0.3), data = list(I = y),
#     lower = 1, upper = 15)
#
# Coefficients:
#      Estimate Std. Error z value     Pr(z)
# beta  4.41228    0.18417  23.957 < 2.2e-16 ***
# ---
# Signif. codes:  0 ¡¥***¡¦ 0.001 ¡¥**¡¦ 0.01 ¡¥*¡¦ 0.05 ¡¥.¡¦ 0.1 ¡¥ ¡¦ 1
#
```

```
#
#    2.5 %   97.5 %
# 4.061687 4.783852



###########different roomp =rp=0.3 ###################################
################# aratio=0.465 #######################################
roomp =0.3
betacand = seq(0.1,15, by = 0.05)
ll = rep(NA, length(betacand))

# plot the likelihood and check maximum
for(i in 1:length(betacand)){
     ll[i] = llik.cb.new(beta = betacand[i],
     I = y, aratio=0.465, roomp=0.3)}
plot(ll ~ betacand, ylab = "Neg log-lik",
     xlab = expression(beta))
betacand[which.min(ll)]

# > betacand[which.min(ll)]
# [1] 4.3

fit = mle2(llik.cb.new, start = list(beta = 4.3), fixed=list(aratio=0.465, roomp=0.3),
    method = "Brent", data = list(I = y), lower=1, upper=15)


summary(fit)
confint(fit)

# > summary(fit)
# Maximum likelihood estimation
#
# Call:
#  mle2(minuslogl = llik.cb.new, start = list(beta = 4.3), method = "Brent",
#     fixed = list(aratio = 0.465, roomp = 0.3), data = list(I = y),
#     lower = 1, upper = 15)
#
# Coefficients:
#      Estimate Std. Error z value     Pr(z)
# beta  4.27816    0.17823  24.004 < 2.2e-16 ***
# ---
# Signif. codes:  0 ¡¥***¡¦ 0.001 ¡¥**¡¦ 0.01 ¡¥*¡¦ 0.05 ¡¥.¡¦ 0.1 ¡¥ ¡¦ 1
#
# -2 log L: 492.5544
# > confint(fit)
# Profiling...
#
#    2.5 %   97.5 %
#  3.938852 4.637700



###########different roomp =rp=0.3 ###################################
################# aratio=0.505 #######################################
roomp =0.3
betacand = seq(0.1,15, by = 0.05)
ll = rep(NA, length(betacand))

# plot the likelihood and check maximum
for(i in 1:length(betacand)){
     ll[i] = llik.cb.new(beta = betacand[i],
     I = y, aratio=0.505, roomp=0.3)}
plot(ll ~ betacand, ylab = "Neg log-lik",
     xlab = expression(beta))
betacand[which.min(ll)]

# > betacand[which.min(ll)]
```

```
    method = "Brent", data = list(I = y), lower=1, upper=15)


summary(fit)
confint(fit)
# > summary(fit)
# Maximum likelihood estimation
#
# Call:
# mle2(minuslogl = llik.cb.new, start = list(beta = 4.2), method = "Brent",
#     fixed = list(aratio = 0.505, roomp = 0.3), data = list(I = y),
#     lower = 1, upper = 15)
#
# Coefficients:
#      Estimate Std. Error z value     Pr(z)
# beta  4.19985    0.17477  24.031 < 2.2e-16 ***
# ---
# Signif. codes:  0 ¡¥***¡¦ 0.001 ¡¥**¡¦ 0.01 ¡¥*¡¦ 0.05 ¡¥.¡¦ 0.1 ¡¥ ¡¦ 1
#
# -2 log L: 457.5956
# > confint(fit)
# Profiling...
#
#    2.5 %   97.5 %
#  3.867103 4.552382



###########different roomp =rp=0.3 ###################################
################# aratio=0.6 #########################################
roomp =0.3
betacand = seq(0.1,15, by = 0.05)
ll = rep(NA, length(betacand))

# plot the likelihood and check maximum
for(i in 1:length(betacand)){
     ll[i] = llik.cb.new(beta = betacand[i],
     I = y, aratio=0.6, roomp=0.3)}
plot(ll ~ betacand, ylab = "Neg log-lik",
     xlab = expression(beta))
betacand[which.min(ll)]

# > betacand[which.min(ll)]
# [1] 4.05

fit = mle2(llik.cb.new, start = list(beta = 4.05), fixed=list(aratio=0.6, roomp=0.3),
    method = "Brent", data = list(I = y), lower=1, upper=15)


summary(fit)
confint(fit)

# > summary(fit)
# Maximum likelihood estimation
#
# Call:
# mle2(minuslogl = llik.cb.new, start = list(beta = 4.05), method = "Brent",
#     fixed = list(aratio = 0.6, roomp = 0.3), data = list(I = y),
#     lower = 1, upper = 15)
#
# Coefficients:
#      Estimate Std. Error z value     Pr(z)
# beta  4.02549    0.16709  24.091 < 2.2e-16 ***
# ---
# Signif. codes:  0 ¡¥***¡¦ 0.001 ¡¥**¡¦ 0.01 ¡¥*¡¦ 0.05 ¡¥.¡¦ 0.1 ¡¥ ¡¦ 1
#
# -2 log L: 389.1513
# > confint(fit)
```

```
# 3.707301 4.362493



######################################################################
######################################################################
################# R0, aratio=0.505 rp=0.2 ############################
beta<- 3.78
ubeta<- 4.09
lbeta<- 3.49
betaci<- c(lbeta, ubeta)

# R0 at time 3
N0[1]*(1-exp(-beta/N0[1]))
# > N0[1]*(1-exp(-beta/N0[1]))
#        0
# 3.778076

#> N0[1]*(1-exp(-betaci/N0[1]))
# [1] 3.488359 4.087747


# R0  t=4 crew
c0[4] *(1-exp(-beta/N0[3]))
# > c0[4] *(1-exp(-beta/N0[3]))
#       3
# 1.059453

c0[4] *(1-exp(-betaci/N0[3]))
# > c0[4] *(1-exp(-betaci/N0[3]))
# [1] 0.9782106 1.1462913


# passengers in cabins t=4 with infected case
# transmission prob
(1-exp(-beta/2))
#> (1-exp(-beta/2))
#[1] 0.8489282


# R0 for passengers in cabins with contacts with asymptomatic crew
# t=4
(pgr0[4]-ipger[4])*(1-exp(-beta*0.505/c0[4]))
# > (pgr0[4]-ipger[4])*(1-exp(-beta*0.505/c0[4]))
#       3
# 4.730159

(pgr0[4]-ipger[4])*(1-exp(-betaci*0.505/c0[4]))
# > (pgr0[4]-ipger[4])*(1-exp(-betaci*0.505/c0[4]))
# [1] 4.367571 5.117696

# overall R0 for both crew and passengers at t=4
R4sum<- c0[4] *(1-exp(-beta/N0[3]))+(pgr0[4]-ipger[4])*(1-exp(-beta*0.505/c0[4]))
#> c0[4]+(pgr0[4]-ipger[4])
#   3
#3609

beta4<- 3609*(-log(1-R4sum/3609))/2
#> beta4
#      3
#2.89713


R4sum<- c0[4] *(1-exp(-betaci/N0[3]))+(pgr0[4]-ipger[4])*(1-exp(-betaci*0.505/c0[4]))
beta4ci<- 3609*(-log(1-R4sum/3609))/2
#> beta4ci
#[1] 2.674873 3.134715
```

```
# > c0[5] *(1-exp(-beta/N0[4]))
#       4
# 1.051642

c0[5] *(1-exp(-betaci/N0[4]))
# > c0[5] *(1-exp(-betaci/N0[4]))
# [1] 0.9709987 1.1378391

# R0 for passengers in cabins with contacts with asymptomatic crew
# t=5
(pgr0[5]-ipger[5])*(1-exp(-beta*0.505/c0[5]))
#> (pgr0[5]-ipger[5])*(1-exp(-beta*0.505/c0[5]))
#       4
# 4.394232


(pgr0[5]-ipger[5])*(1-exp(-betaci*0.505/c0[5]))
#> (pgr0[5]-ipger[5])*(1-exp(-betaci*0.505/c0[5]))
#[1] 4.057401 4.754239

#overall R0 for both crew and passengers at t=5
R5sum<- c0[5] *(1-exp(-beta/N0[4]))+(pgr0[5]-ipger[5])*(1-exp(-beta*0.505/c0[5]))
#> c0[5]+(pgr0[5]-ipger[5])
#   3
# 3357

beta5<- 3357*(-log(1-R5sum/3357))/2
#> beta5
#       4
#2.725148

R5sum<- c0[5] *(1-exp(-betaci/N0[4]))+(pgr0[5]-ipger[5])*(1-exp(-betaci*0.505/c0[5]))
beta5ci<- 3357*(-log(1-R5sum/3357))/2
#> beta5ci
#[1] 2.516084 2.948628


######################################################################
####    PLOT figure 1 (a) ############################################
par(mfrow=c(1,2))
plot(x=c(1, 18, 19, 23, 24, 30), y=c(3.78, 3.78, 4.73, 4.73, 4.39, 4.39), col=4, ylim=c(0, 5), type="l", xlab
main="serial interval=6 days")
lines(x=c(1, 18, 19, 23, 24,  30), y=c(3.49, 3.49, 4.37, 4.37, 4.06, 4.06), lty=2, col=4)
lines(x=c(1, 18, 19, 23, 24,  30), y=c(4.09, 4.09, 5.12, 5.12, 4.75, 4.75), lty=2, col=4)

lines(x=c(1, 18, 19, 23, 24,  30), y=c(3.78, 3.78, 1.06, 1.06, 1.05, 1.05), col=2)
lines(x=c(1, 18, 19, 23, 24,  30), y=c(3.49, 3.49, 0.98, 0.98, 0.97, 0.97), lty=2, col=2)
lines(x=c(1, 18, 19, 23, 24,  30), y=c(4.09, 4.09, 1.15, 1.15, 1.14, 1.14), lty=2, col=2)

lines(x=c(1,18), y=c(3.78, 3.78))
lines(x=c(1, 18), y=c(3.49, 3.49), lty=2)
lines(x=c(1,  18), y=c(4.09, 4.09), lty=2)

######################################################################
######### PLOT figure 1(b) ###########################################
############# PLOT serial interval=5 days ##############################

plot(x=c(1, 15, 16, 20, 21, 25, 26, 30), y=c(3.27, 3.27, rep(4.18,2), rep(4.08,2), rep(3.74,2)), col=4, ylim=c
xlab="Day", ylab="R0", main="serial interval=5 days")
lines(x=c(1, 15, 16, 20, 21, 25, 26, 30), y=c(rep(3.02,2), rep(3.86,2), rep(3.77, 2), rep(3.45,2)), lty=2, co
lines(x=c(1, 15, 16, 20, 21, 25, 26, 30), y=c(rep(3.54,2), rep(4.52,2), rep(4.42, 2), rep(4.04,2)), lty=2, co

lines(x=c(1, 15, 16, 20, 21, 25, 26, 30), y=c(3.27, 3.27, rep(0.92, 4), rep(0.91,2)), col=2)
lines(x=c(1, 15, 16, 20, 21, 25, 26, 30), y=c(rep(3.02,2), rep(0.85, 4), rep(0.84,2)), lty=2, col=2)
lines(x=c(1, 15, 16, 20, 21, 25, 26, 30), y=c(rep(3.54,2), rep(1,2), rep(0.99,2), rep(0.98,2)), lty=2, col=2)

lines(x=c(1,15), y=c(3.27, 3.27))
lines(x=c(1, 15), y=c(3.02, 3.02), lty=2)
```

```
######################################################################

######################################################################
################# R0, aratio=0.505 rp=0.3 ############################
beta<- 4.20
ubeta<- 3.87
lbeta<- 4.55
betaci<- c(lbeta, ubeta)

# R0 at t=3
N0[1]*(1-exp(-beta/N0[1]))
# > N0[1]*(1-exp(-beta/N0[1]))
#        0
# 4.197624

# > N0[1]*(1-exp(-betaci/N0[1]))
# [1]  4.547212 3.867983


# R0  t=4 for crew
c0[4] *(1-exp(-beta/N0[3]))
# > c0[4] *(1-exp(-beta/N0[3]))
#        3
# 1.177103

c0[4] *(1-exp(-betaci/N0[3]))
# > c0[4] *(1-exp(-betaci/N0[3]))
# [1] [1] 1.275135 1.084665


# passengers in cabins t=4 with infected case
#transmission prob
(1-exp(-beta/2))
#> (1-exp(-beta/2))
#[1]  0.8775436


# R0 for passengers in cabins with contacts with asymptomatic crew#
# t=4
(pgr0[4]-ipger[4])*(1-exp(-beta*0.505/c0[4]))
#> (pgr0[4]-ipger[4])*(1-exp(-beta*0.505/c0[4]))
#        3
# 5.255195


(pgr0[4]-ipger[4])*(1-exp(-betaci*0.505/c0[4]))
# > (pgr0[4]-ipger[4])*(1-exp(-betaci*0.505/c0[4]))
# [1]  5.692643 4.842676


#overall R0 for both crew and passengers t=4
R4sum<- c0[4] *(1-exp(-beta/N0[3]))+(pgr0[4]-ipger[4])*(1-exp(-beta*0.505/c0[4]))
#> R4sum
#        3
# 6.432298
# > c0[4]+(pgr0[4]-ipger[4])
#    3
# 3609

beta4<- 3609*(-log(1-R4sum/3609))/2
#> beta4
#       3
# 3.219018


R4sum<- c0[4] *(1-exp(-betaci/N0[3]))+(pgr0[4]-ipger[4])*(1-exp(-betaci*0.505/c0[4]))
beta4ci<- 3609*(-log(1-R4sum/3609))/2
# > beta4ci
```

```
c0[5] *(1-exp(-beta/N0[4]))
#> c0[5] *(1-exp(-beta/N0[4]))
#       4
#1.168424


c0[5] *(1-exp(-betaci/N0[4]))
#> c0[5] *(1-exp(-betaci/N0[4]))
#[1] 1.265731 1.076667

# R0 for passengers in cabins with contacts with asymptomatic crew#
# t=5
(pgr0[5]-ipger[5])*(1-exp(-beta*0.505/c0[5]))
#> (pgr0[5]-ipger[5])*(1-exp(-beta*0.505/c0[5]))
#       4
# 4.88197


(pgr0[5]-ipger[5])*(1-exp(-betaci*0.505/c0[5]))
# > (pgr0[5]-ipger[5])*(1-exp(-betaci*0.505/c0[5]))
# [1]  5.288341 4.498756

# overall R0 for both crew and passengers t=5

R5sum<- c0[5] *(1-exp(-beta/N0[4]))+(pgr0[5]-ipger[5])*(1-exp(-beta*0.505/c0[5]))
#> c0[5]+(pgr0[5]-ipger[5])
#   3
# 3357

beta5<- 3357*(-log(1-R5sum/3357))/2
# > beta5
#       4
# 3.027926


R5sum<- c0[5] *(1-exp(-betaci/N0[4]))+(pgr0[5]-ipger[5])*(1-exp(-betaci*0.505/c0[5]))
beta5ci<- 3357*(-log(1-R5sum/3357))/2
# > beta5ci
# [1] 3.280240 2.790029


######################################################################
######################################################################

##########  PLOT Figure 2   ###########################################

set.seed(20200302)
plot(y, type="n", xlim=c(0,16), ylim=c(1, 1800),
   ylab="Predicted/observed cases", xlab="time in units of the serial interval of 6 days")

#here the first j index correspond1 to time 0
It<- matrix(1, nrow=100, ncol=16)
newI<- matrix(0, nrow=100, ncol=16 )
newI[,1]<- 1
Sus <- matrix(3700, nrow=100, ncol=16)


for (i in 1:100) {
   Ntotal<-3700
   for (j in 2:16) {
        if (Sus[i,j-1] >0)
        { newcase=rbinom(1, size=Sus[i,j-1], prob=(1-exp(-3.78*newI[i,j-1]/Sus[i,j-1])))
          newI[i,j]=newcase
          It[i,j]=It[i,j-1]+newcase
        }
        if (Sus[i,j-1]> newcase) { Sus[i,j]=Sus[i,j-1]-newcase
                                   Ntotal=Ntotal-newcase  }
        else Sus[i,j]=0
```

```
  }
points(seq(0, 5, by=1),y, type="b", col=2)



##################################################################################
#
#
#> summary(It[,6])
#   Min. 1st Qu.  Median    Mean 3rd Qu.    Max.
#    1.0   562.0   790.5   855.7  1100.0  2382.0
#
#> sqrt(var(It[,6]))
#[1] 439.6533
#
#########################
#> summary(Sus[,11])
#   Min. 1st Qu.  Median    Mean 3rd Qu.    Max.
#   2.00    9.75   12.00   85.71   16.00 1480.00
#> summary(Sus[,6])
#   Min. 1st Qu.  Median    Mean 3rd Qu.    Max.
#  440.0   723.8   895.0   905.6  1107.2  1480.0

######################################################################
################### The END ##########################################
```
